# Genetic Variants Influencing Biomarkers of Nutrition Are Not Associated with Cognitive Capability in Middle-Aged and Older Adults^1^,^2^,^3^

**DOI:** 10.3945/jn.112.171520

**Published:** 2013-03-06

**Authors:** Tamuno Alfred, Yoav Ben-Shlomo, Rachel Cooper, Rebecca Hardy, Ian J. Deary, Jane Elliott, Sarah E. Harris, Elina Hyppönen, Mika Kivimaki, Meena Kumari, Jane Maddock, Chris Power, John M. Starr, Diana Kuh, Ian N.M. Day, the HALCyon Study Team

**Affiliations:** 5 School of Social and Community Medicine, University of Bristol, Bristol, UK; 6 MRC Unit for Lifelong Health and Ageing and Division of Population Health, University College London, London, UK; 7 Centre for Cognitive Ageing and Cognitive Epidemiology, and; 8 Department of Psychology, University of Edinburgh, Edinburgh, UK; 9 Centre for Longitudinal Studies, Department of Quantitative Social Sciences, Institute of Education, London, UK; 10 Medical Genetics Section, University of Edinburgh, Edinburgh, UK; 11 MRC Centre of Epidemiology for Child Health/Centre for Paediatric Epidemiology and Biostatistics, UCL Institute of Child Health, London, UK; 12 Department of Epidemiology and Public Health, University College London, London, UK; 13 Alzheimer Scotland Dementia Research Centre, University of Edinburgh, Edinburgh, UK; and; 14 MRC Centre for Causal Analyses in Translational Epidemiology, School of Social and Community Medicine, University of Bristol, Bristol, UK

## Abstract

Several investigations have observed positive associations between good nutritional status, as indicated by micronutrients, and cognitive measures; however, these associations may not be causal. Genetic polymorphisms that affect nutritional biomarkers may be useful for providing evidence for associations between micronutrients and cognitive measures. As part of the Healthy Ageing across the Life Course (HALCyon) program, men and women aged between 44 and 90 y from 6 UK cohorts were genotyped for polymorphisms associated with circulating concentrations of iron [rs4820268 transmembrane protease, serine 6 (*TMPRSS6*) and rs1800562 hemochromatosis (*HFE*)], vitamin B-12 [(rs492602 fucosyltransferase 2 (*FUT2*)], vitamin D ([rs2282679 group-specific component (*GC*)] and β-carotene ([rs6564851 beta-carotene 15,15'-monooxygenase 1 (*BCMO1*)]. Meta-analysis was used to pool within-study effects of the associations between these polymorphisms and the following measures of cognitive capability: word recall, phonemic fluency, semantic fluency, and search speed. Among the several statistical tests conducted, we found little evidence for associations. We found the minor allele of rs1800562 was associated with poorer word recall scores [pooled β on Z-score for carriers vs. noncarriers: −0.05 (95% CI: −0.09, −0.004); *P* = 0.03, *n* = 14,105] and poorer word recall scores for the vitamin D–raising allele of rs2282679 [pooled β per T allele: −0.03 (95% CI: −0.05, −0.003); *P* = 0.03, *n* = 16,527]. However, there was no evidence for other associations. Our findings provide little evidence to support associations between these genotypes and cognitive capability in older adults. Further investigations are required to elucidate whether the previous positive associations from observational studies between circulating measures of these micronutrients and cognitive performance are due to confounding and reverse causality.

## Introduction

Adequate nutrition is essential for good health throughout the life course, and low-quality diets in later life have been associated with adverse outcomes, such as increased risk of coronary heart disease (1) and mortality (2, 3). Measures of dietary intake of specific micronutrients, along with their biomarkers, have also been associated with indicators of health in observational epidemiologic studies and there is evidence from many, although not all, studies that micronutrients are important to cognitive outcomes (4, 5). Iron deficiency anemia is negatively associated with cognitive performance in childhood (6), and positive associations between cognitive measures and several other micronutrients have also been observed in adults including circulating concentrations of vitamin B-12 (7) and vitamin D (8, 9) and intake (10) and plasma concentrations of β-carotene (11).

The associations observed between nutrition and cognitive measures may not, however, be causal in all cases. Animal studies have shown that a diet rich in antioxidants may delay cognitive aging (12), studies in children with iron deficiency anemia show iron supplementation may improve cognition (6), and there is evidence for improvements in cognition from some, although not all, intervention studies for nutrients in adults (13). Whereas mixed evidence from intervention studies may be partly due to inadequately short treatment lengths for the observation of effects on cognitive measures, there are other possible noncausal explanations for the associations observed between nutrients and cognitive measures. For example, effects may be due to the lifestyle factors that have been associated with the intake of some nutrients (14) or due to cognitive impairment leading to reduced nutrient intake (4). Genetic variants associated with biomarkers of nutrition may be useful in providing evidence for associations with cognitive measures. Any associations would be less likely to be due to confounding (15) or reverse causality (16). The assumptions for this hypothesis are presented in **Supplemental Fig. 1**. Furthermore, genetic variants implicated in the circulating concentrations of biomarkers of nutrition may be additionally informative about the long-term effects of lower concentrations.

We therefore investigated the associations between genetic variants implicated in the concentrations of biomarkers of nutrition, from genome-wide association studies (GWASs)^15^, and measures of cognitive capability, the capacity to undertake the mental tasks of daily living, in 20,528 participants aged between 44 and 90 y as part of the Healthy Ageing across the Life Course (HALCyon) (17) collaborative research program. Although micronutrient biomarkers may not be direct measures of dietary intake in generally well-nourished individuals, they are important indicators of deficiency and storage.

Serum iron is ∼23% heritable (18), and increased concentrations have been associated with the A allele of the single nucleotide polymorphism (SNP) rs4820268 in transmembrane protease, serine 6 (*TMPRSS6*) (19, 20) and with the A allele of rs1800562 (C282Y) in hemochromatosis (*HFE*) (21), the former being common in many populations, whereas homozygosity of the latter is present in <0.5% of individuals of European ancestry (22) and is found in 80% of individuals with hemochromatosis (23), an iron overload syndrome. The G allele of rs492602 in fucosyltransferase 2 (*FUT2*) has been associated with increased plasma vitamin B-12 (24). SNPs that contribute to the estimated 28% heritability of concentrations of 25-hydroxyvitamin D in individuals of European ancestry (25) have also been identified, with the T allele of rs2282679 [group-specific component (*GC*)] associated with higher concentrations (26, 27). The G allele of SNP rs6564851 near beta-carotene 15,15'-monooxygenase 1 (*BCMO1*) has been associated with higher β-carotene, an antioxidant precursor to vitamin A (28). We hypothesized that these variants, identified from GWASs, would be associated with measures of cognitive capability, such as verbal memory and fluency, in our studies in middle-aged and older adults.

## Materials and Methods

### Study populations

The National Child Development Study (NCDS) has followed up all individuals born in England, Scotland, and Wales during 1 wk in March 1958. In 2002–2004 a biomedical survey was conducted during home visits by a research nurse. After informed consent, DNA was extracted from 8017 participants aged 44–45 y; the sample with the immortalized cell line culture (*n* = 7526) is used here. In 2008–2009, an eighth sweep was carried out during which cognitive performance tests were conducted (29). Further details of the study are available elsewhere (30). The Medical Research Council National Survey of Health and Development (NSHD) comprises participants sampled from all births in a week in March 1946 and followed up since then. In 1999, at age 53 y, men and women were visited by a research nurse and consent for DNA extraction was given by ∼2900 members of the cohort. Details of the data collected and the several phases of the study are available on the cohort’s website (31) and elsewhere (32).

The Whitehall II study targeted all civil servants aged between 35 and 55y working in London in 1985–1988. In 2002–2004 (phase VII), the genetics study was established and DNA was extracted from 6156 participants. Details of the study design and data collected have been described elsewhere (33).

The Caerphilly Prospective Study (CaPS) recruited 2512 men aged between 45 and 59 y in 1979–1983 from the town of Caerphilly, South Wales, and its surrounding villages. Blood samples were collected at baseline and at each of the 4 follow-ups (phase II: 1984–1988; phase III: 1989–1993; phase IV: 1993–1997; and phase V: 2002–2004.) Further details are available on the cohort’s website (34).

The English Longitudinal Study of Ageing (ELSA) comprises men and women aged ≥50 y who originally participated in the Health Survey for England in 1998, 1999, or 2001. Fieldwork began in 2002–2003 (phase I) with biennial follow-ups, and in 2004–2005 (phase II) blood samples were provided by 6231 participants. Details of the cohort have been published elsewhere (35).

The Lothian Birth Cohort 1921 (LBC1921) participants were all born in 1921, and most completed a cognitive ability assessment at age 11 y. In 1999–2001 (wave I), at age ∼79 y, 550 participants living in and around Edinburgh underwent a series of cognitive and physical tests. Details of the recruitment into the study are available on its website (36) and have been published previously (37, 38).

Informed consent was obtained from all participants. Ethical approval was obtained from the South-east Multi-center Research Ethics Committee, the North Thames Multi-center Research Ethics Committee, the Joint University College London/University College London Hospitals Committees on the Ethics of Human Research (Committee Alpha), the Medical Research and Ethics Committee, and the Lothian Research Ethics Committee.

### Genotyping and quality control

Genotyping for all SNPs [rs4820268 (*TMPRSS6*, 22q12.3), rs1800562 (C282Y, *HFE*, 6p22.2), rs492602 (*FUT2*, 19q13.33), rs2282679 (*GC*, 4q13.3), and rs6564851 (near *BCMO1*, 16q23.2)] in CaPS and ELSA were carried out by KBioscience (39) along with rs492602 in NCDS and rs2282679 in NSHD. Genotype information for the remaining SNPs in NSHD and all SNPs in Whitehall II came from the Illumina Metabochip (40). Genotype information for all SNPs in LBC1921 came from the Illumina Human 610-Quadv1 Chip (41), with rs492602 imputed by using the HapMap phase II CEU data [National Center for Biotechnology Information build 36 (UCSC hg18)] as the reference sample and MACH software; imputation quality score was high (*r*^2^ = 0.99). The remaining SNPs in NCDS came from 2 sources: Illumina HumanHap 550k v3 and Illumina 1.2M chips (42). Genotypic data quality was reviewed by assessing departure from the Hardy-Weinberg equilibrium, clustering quality (using KBioscience software SNPviewer on their data) and call rates.

### Phenotypes

#### Cognitive capability.

A number of cognitive performance tests in the different studies were used to assess cognitive capability. Different assessments of verbal memory were conducted: in ELSA and NCDS, a list of 10 common words were used with participants asked to recall the list immediately and again after a delay (the mean score was used in the analysis); in NSHD, 15 words were used over 3 trials; in Whitehall II, 20 words were used. Responses in NSHD and Whitehall II were given in writing. In Whitehall II, participants recalled in writing in 1 min as many words as possible beginning with “S” to assess phonemic fluency, whereas in LBC1921 the 3 letters “C,” “F,” and “L” were used with responses given orally. Participants were asked to recall as many animals as possible within 1 min to measure semantic fluency; responses were given orally in ELSA, NCDS, CaPS, and NSHD and in writing in Whitehall II. To assess search speed (43), 1-min letter searches among grids of letters were used: 600 letters in NSHD and 780 in ELSA and NCDS.

#### Anthropometric measurements.

BMI (kg/m^2^) was calculated as weight divided by height squared derived from measurements conducted at clinics, during a clinical interview in the home, or from self-report. Waist-hip ratio was defined as waist circumference (cm) divided by hip circumference (cm).

#### Demographic characteristics.

Demographic information was derived from self-report. Where information on ethnicity was collected, participants of non-European ancestry were excluded from analyses to avoid confounding from population stratification (44). Levels of physical activity were derived from questionnaires. Individuals were categorized as “physically active” in this analysis if they engaged, at least once a month, in at least moderate sport or activities in NCDS, NSHD, CaPS, and LBC1921 or in vigorous sport or activities in ELSA and Whitehall II. Participants’ alcohol consumption was dichotomized here into “at least weekly” and “less often” in all studies, except for NSHD, where “more often than special occasions” and “less often” were used. Data on current smoking status and socioeconomic position were also used.

### Statistical methods

Within studies, linear and logistic regression analyses were conducted on the continuous and dichotomous traits within the cohorts respectively, adjusting for sex in all studies except for CaPS and for age in all studies except for those that were age homogenous (i.e., NCDS, NSHD, and LBC1921). Adjustments for anthropometric measures in the cognitive capability models were made where appropriate. NCDS and NSHD were used to additionally adjust the genotypic effects of rs4820268 for glycated hemoglobin (Hb A_1c_), and of rs1800562 for Hb A_1c_ and LDL cholesterol in the cognitive capability models, due to evidence for associations with these traits (45, 46). Dominant models were used for rs1800562 due to the low frequency of individuals homozygous for the minor alleles (*n* = 92; 0.6%). Additive models for the other SNPs were used with genotypes coded as 0, 1, and 2 for the number of biomarker-raising alleles. Likelihood ratio tests were used to compare the fit of the additive models compared with the full genotype model for the cognitive capability traits. For continuous traits, the normality of the standardized residuals was inspected with distributional diagnostic plots. For the harmonization of continuous traits that were used to obtain pooled estimates of the genotypic effects, Z-score units were calculated in each study by subtracting the study mean and dividing by its SD. The overall mean for Z-scores is 0 and the SD is 1. Two-step (47) meta-analyses using a random-effects model were performed to obtain pooled genotypic effects. The *I*^2^ measure was used to quantify heterogeneity (48). Finally, the calculation of Z-scores, for continuous traits, and the main analyses were repeated in men and women separately. Reporting of the analyses met the appropriate items of recommended checklists (49, 50). A 2-tailed significance level of *P* < 0.05 was used as evidence of statistical significance. Statistical analysis was performed in Stata 11.2 (StataCorp LP).

## Results

### 

#### Cohort summaries and genotyping quality.

**Table 1** shows that a total of 20,528 men and women aged between 44 and 90 y had relevant genotypic and phenotypic data. Summary measures of the anthropometric and demographic variables in the cohorts are presented in **Supplemental Table 1**. Call rates were high, exceeding 94% for all SNPs across the studies. The Hardy-Weinberg equilibrium condition was met for all SNPs in both sources of NCDS and in all other studies (*P* > 0.06).

**TABLE 1:** Summary of sex, age, and nutrient biomarker–raising allele frequencies by cohort^1^

	Cohort						
Characteristic	NCDS	NSHD	Whitehall II	CaPS	ELSA	LBC1921	Total
Participants,^2^ *n*	7386	2649	3143	1224	5613	513	20,528
Male, *%*	50	50	77	100	46	41	56
Age,^3^ *y*	44	53	59 (50 to 73)	62 (52 to 71)	65 (52 to ≥90)	79 (77 to 80)	53 (44 to ≥90)
Biomarker-raising allele frequency							
rs4820268 *TMPRSS6*, A	0.54	0.53	0.54	0.52	0.54	0.58	0.54
rs1800562 *HFE*, A	0.08	0.08	0.07	0.07	0.07	0.10	0.08
rs492602 *FUT2*, G	0.51	0.51	0.51	0.51	0.50	0.56	0.51
rs2282679 *GC*, T	0.71	0.72	0.71	0.72	0.71	0.71	0.71
rs6564851 *BCMO1*, G	0.53	0.55	0.54	0.54	0.53	0.57	0.54

1 *BCMO1*, beta-carotene 15,15'-monooxygenase 1; CaPS, Caerphilly Prospective Study; ELSA, English Longitudinal Study of Ageing;* FUT2*, fucosyltransferase 2; *GC*, group-specific component; *HFE*, hemochromatosis; LBC1921, Lothian Birth Cohort 1921; NCDS, National Child Development Study; NSHD, National Survey of Health and Development;* TMPRSS6*, transmembrane protease, serine 6.

2 Numbers of participants represent those with available data for ≥1 cognitive capability phenotype and ≥1 genotype.

3 Age is presented as median (range) and is age at the phase at which the majority of variables are taken, i.e., NCDS, Biomedical Survey (2002); NSHD, 1999 Collection; Whitehall II, phase VII; CaPS, phase III; ELSA, phase II; LBC1921, phase I.

#### Associations between genotype and phenotypes.

Associations between the genotypes and measures of anthropometry and demographic factors are presented in **Supplemental Tables 2–4**. There was some evidence that the A allele of rs4820268 (*TMPRSS6*) was associated with lower weight (*P* = 0.02) and BMI (*P* = 0.01) (Supplemental Table 2). There was no evidence for other genotypic associations for the anthropometric measures or for physical activity, smoking status, alcohol consumption, or socioeconomic position (*P* > 0.07).

Summaries of the pooled genotypic associations with the cognitive capability measures are presented in **Table 2**. A negative association between the A allele of rs4820268 (*TMPRSS6*) and phonemic fluency was observed in LBC1921, but not in Whitehall II, resulting in substantial heterogeneity (*I*^2^ = 83.1%, *P* = 0.02; **Supplemental Fig. 2**, **Supplemental Table 5**), and there was no overall effect, after adjusting for age, sex, height, and weight. There was some evidence that carriers of the A allele of rs1800562 (*HFE*) had lower word recall scores after adjusting for age and sex (*P* = 0.03), as well as for a trend toward slower search speeds (*P* = 0.08; **Supplemental Fig. 3**, **Supplemental Table 6**). We also observed trends toward associations between the G allele of rs492602 (*FUT2*) and poorer performance in semantic fluency (*P* = 0.06) and search speed (*P* = 0.05) (**Supplemental Table 7** and **Supplemental Fig. 4**). Table 2 shows that there was some evidence that the T allele of rs2282679 (*GC*) was associated with lower word recall scores after adjusting for age and sex (*P* = 0.03), although no other pooled associations were observed for this SNP. Differences were observed for the genotypic effects of rs2282679 (*GC*) on phonemic fluency between LBC1921 and Whitehall II (*I*^2^ = 80.6%, *P* = 0.02), with a negative effect of the T allele observed only in LBC12921 (**Supplemental Fig. 5**, **Supplemental Table 8**). A positive association between the G allele of rs6564851 (*BCMO1*) and word recall was observed only in NSHD, with no overall effect from all 4 studies (heterogeneity: *I*^2^ = 70.5%, *P* = 0.02; Table 2, **Supplemental Table 9**, and **Supplemental Fig. 6**).

**TABLE 2:** Summary of pooled associations between genotypes and cognitive capability^1^

Cognitive capability measure and genotype	*n*	β (95% CI)	*P*	*I*^2^(%); *P*-heterogeneity
Word recall				
rs4820268 (*TMPRSS6*)	16,034	−0.003 (−0.024, 0.018)	0.75	1.7; 0.38
rs1800562 (*HFE*)	14,105	−0.049 (−0.095, -0.004)	0.033	0.0; 0.74
rs492602 (*FUT2*)	18,295	−0.003 (−0.023, 0.017)	0.75	0.0; 0.71
rs2282679 *(GC*)	16,527	−0.026 (−0.048, -0.003)	0.028	0.0; 0.73
rs6564851 (*BCMO1*)	16,458	0.021 (−0.018, 0.060)	0.29	70.5; 0.017
Phonemic fluency				
rs4820268 (*TMPRSS6*)	3622	−0.078 (−0.240, 0.085)	0.35	83.1; 0.0151
rs1800562 (*HFE*)	3638	0.046 (−0.044, 0.136)	0.32	0.0; 0.57
rs492602 (*FUT2*)	3639	−0.004 (−0.048, 0.041)	0.88	0.0; 0.45
rs2282679 *(GC*)	3625	−0.045 (−0.205, 0.114)	0.58	80.6; 0.023
rs6564851 (*BCMO1*)	3638	−0.042 (−0.116, 0.033)	0.27	36.6; 0.21
Semantic fluency				
rs4820268 (*TMPRSS6*)	17,311	0.002 (−0.023, 0.026)	0.90	27.5; 0.24
rs1800562 (*HFE*)	15,392	−0.007 (−0.051, 0.036)	0.74	0.0; 0.66
rs492602 (*FUT2*)	19,599	−0.018 (−0.038, 0.001)	0.064	0.0; 0.94
rs2282679 *(GC*)	17,827	−0.010 (−0.046, 0.026)	0.59	56.3; 0.057
rs6564851 (*BCMO1*)	17,741	−0.007 (−0.032, 0.017)	0.57	27.7; 0.24
Search speed				
rs4820268 (*TMPRSS6*)	12,821	0.019 (−0.005, 0.042)	0.12	0.0; 0.73
rs1800562 (*HFE*)	10,910	−0.049 (−0.105, 0.006)	0.08	12.1; 0.32
rs492602 (*FUT2*)	15,039	−0.026 (−0.052, 0.000)	0.053	25.9; 0.26
rs2282679 *(GC*)	13,305	−0.003 (−0.029, 0.023)	0.84	0.0; 0.87
rs6564851 (*BCMO1*)	13,213	0.008 (−0.015, 0.031)	0.50	0.0; 0.42

1 Coefficients are based on Z-scores. All coefficients were adjusted for age and sex; rs4820268 was additionally adjusted for height and weight. rs4820268, per A allele; rs1800562, A/A + A/G vs. G/G; rs492602, per G allele; rs2282679, per T allele; rs6564851, per G allele.* BCMO1*, beta-carotene 15,15'-monooxygenase 1 ; *FUT2*, fucosyltransferase 2; *GC*, group-specific component; *HFE*, hemochromatosis; *TMPRSS6*, transmembrane protease, serine 6.

There was no evidence for other genotypic associations for the measures of cognitive capability in the pooled analyses. In only a relatively small number of tests did the full genotype model represent a significantly better fit than the per allele model (indicated in Supplemental Tables 7 and 8). Full genotypic associations are presented in Supplemental Tables 5–9 and Supplemental Figs. 2–6. There was no evidence for heterogeneity between men and women for any of the genotypic associations for any of the cognitive capability measures (*P* > 0.08; data not shown), except for rs6564851 (*BCMO1*) where its effects on search speed differed (*P*-heterogeneity = 0.045), although not achieving significance in either sex (men, *P* = 0.05; women, *P* = 0.41).

Additional adjustment for Hb A_1c_ in the models of effects of rs4820268 (*TMPRSS6*) in NCDS and NSHD did not affect the null associations observed in those studies, nor did the adjustment for LDL cholesterol and Hb A_1c_ for rs1800562 (*HFE*) attenuate the pooled negative association between the A allele and word recall for the 2 studies [(*n* = 7042; pooled β for Z-score: −0.09 (95% CI: −0.16, −0.03); *P* = 0.005, *I*^2^ = 0.0%)], nor markedly affect any of the other associations (data not shown).

## Discussion

We investigated associations between SNPs robustly associated with nutritional biomarkers from GWASs and measures of cognitive capability in 20,528 adults aged between 44 and 90 y from 6 UK cohorts. There was little evidence for important associations, with some evidence that carriers of the serum-raising and hemochromatosis-predisposing allele of rs1800562 (C282Y) in *HFE* performed poorer in word recall tests (*P* = 0.03). We also observed a negative association between the allele of rs2282679 (*GC*) associated with higher vitamin D and word recall (*P* = 0.03), as well as a trend toward poorer performance in semantic fluency and search speed for the allele of rs492602 (*FUT2*), which is associated with higher vitamin B-12 (24). We observed no associations between SNP rs4820268 (*TMPRSS6*), associated with relatively lower effects on serum iron than C282Y (51), or between the SNP associated with β-carotene, rs6564851 (*BCMO1*), on any of our investigated cognitive capability traits. Our findings therefore suggest that the genotypes that affect these nutritional biomarkers have little, if any, association with measures of cognitive capability, including verbal fluency and memory in middle-aged and older adults.

Given the burden of cognitive decline in aging populations (5), inferring causality between biomarkers of nutrition and cognitive measures could have implications for public health policy, and there have been several investigations into associations between dietary intake, or circulating measures, of micronutrients on cognitive measures, with some positive associations observed (4). However, these associations may be confounded by lifestyle factors, be due to better cognition informing better nutritional choices (4), or reflect temporary nutritional status only. There have been fewer investigations using genetic markers implicated in the circulating concentrations, which may help to reduce these effects of confounding and reverse causality and help to investigate the effects of long-term levels of exposure (15, 16). One smaller study in 818 men and women aged between 50 and 70 y observed no association between C282Y (*HFE*) and several cognitive measures (52), and a study in 358 men observed no association between genotypes of *HFE* and performance in the Mini-Mental State Examination, but there was some evidence that the genotypes did modify the effects of lead burden on the change in scores (53). With the emergence of robust genotypic associations identified from GWASs, we were able to investigate the effects of circulating concentrations of serum iron as well as additional markers on cognitive capability. The overall null associations we found between the investigated genotypes associated with nutritional biomarkers and cognitive capability do not support a hypothesis of direct causal effects.

There are, however, various hypothesized mechanisms through which micronutrients may directly affect cognitive capability. Animal studies have shown that iron deficiency leads to an increase in iron uptake in the brain (54), and increased iron content is often seen in the brains of patients with neurodegenerative diseases, including Alzheimer disease (55) and Parkinson disease (56), and it has been suggested that iron deficiency may be implicated in such diseases (57), although its role is not fully understood. Low B vitamin status may also affect brain tissue by impairing necessary homocysteine methylation (58). In addition, higher concentrations of plasma vitamin B-12 have been associated with lower rates of brain volume loss in the elderly (59). The brain’s susceptibility to free radical damage makes antioxidants important actors in its defense from oxidative stress (60) and as a result may also affect cognitive capability.

It is therefore possible that these micronutrients may be able to affect cognitive capability measures, and despite the large size of our study it may still have been underpowered to provide strong evidence for these effects. In addition to this, there are other factors to be considered in the interpretation of results from our investigation. Interpretation of a few of our findings may be hindered by large heterogeneity that we observed and further investigations may explain any possible sources. The inverse association observed between the vitamin D–raising allele and word recall from the pooled results was not seen for any of the other cognitive measures and may therefore be spurious, especially in light of the number of statistical tests conducted. It is possible that the association we observed between the serum iron–raising allele of rs1800562 (C282Y) in *HFE* and poorer word recall is an indicator of any effect of a predisposition to hemochromatosis, a syndrome often accompanied by fatigue and malaise (23), and not directly of serum iron, because this association was not observed for the rs4820268 (*TMPRSS6*) genotype. Residual confounding may be reduced further by the inclusion of several genotypes for each micronutrient (61), possibly providing stronger instruments for investigating causality (16).

Although our study found no strong evidence for associations between these genotypes and cognitive capability, attention should still be given to improving and maintaining the overall quality of diets of older people in order to reduce the risk of morbidity and mortality associated with poor diets (1–3). In addition, there is still a possible role of macronutrients in cognitive performance (4). Maintenance of good nutritional status in older adults is particularly important due to physiologic factors and reduced dietary intake of nutrients that may occur in later life, which contribute to the lower concentrations of nutritional biomarkers observed in older age (4, 62). However, food sources may be preferable to pharmacologic supplements because supplementation of synthetic antioxidants, such as β-carotene, and minerals, such as iron, in generally well-nourished individuals may lead to adverse outcomes, such as increased mortality risk (63, 64).

In conclusion, the results of this large multicohort study using genetic variants of nutritional biomarkers require replication but provide little evidence for associations with cognitive capability in middle-aged and older adults.

## Supplementary Material

Online Supporting Material

## Footnotes

15 Abbreviations used: *BCMO1*, beta-carotene 15,15'-monooxygenase; CaPS, Caerphilly Prospective Study; ELSA, English Longitudinal Study of Ageing; *FUT2*, fucosyltransferase 2; *GC*, group-specific component; GWAS, genome-wide association study; HALCyon, Healthy Ageing across the Life Course; Hb A_1c_, glycated hemoglobin; *HFE*, hemochromatosis; LBC1921, Lothian Birth Cohort 1921; NCDS, National Child Development Study; NHSD, National Survey of Health and Development; SNP, single nucleotide polymorphism; *TMPRSS6*, transmembrane protease, serine 6.
