# Supplementary material for: Genetic Variants Influencing Biomarkers of Nutrition Are Not Associated with Cognitive Capability in Middle-Aged and Older Adults
Source: J Nutr. 2013 Mar 6;143(5):606–12. doi: 10.3945/jn.112.171520 (PMC3738233; doi:10.3945/jn.112.171520)
Supplement: Online Supporting Material [file supp_143_5_606__index.html]

Online Supporting Material 

# Genetic Variants Influencing Biomarkers of Nutrition Are Not Associated with Cognitive Capability in Middle-Aged and Older Adults

## Online Supporting Material

**Files in this Data Supplement:**

- Online Supporting Material - Figures 1-6 and Tables 1-9
