# Supplementary material for: Genetic Variants Influencing Biomarkers of Nutrition Are Not Associated with Cognitive Capability in Middle-Aged and Older Adults
Source: J Nutr. 2013 Mar 6;143(5):606–12. doi: 10.3945/jn.112.171520 (PMC3738233; doi:10.3945/jn.112.171520)
Supplement: Online Supporting Material [file jn.112.171520_nutrition171520SupplementaryData.pdf]

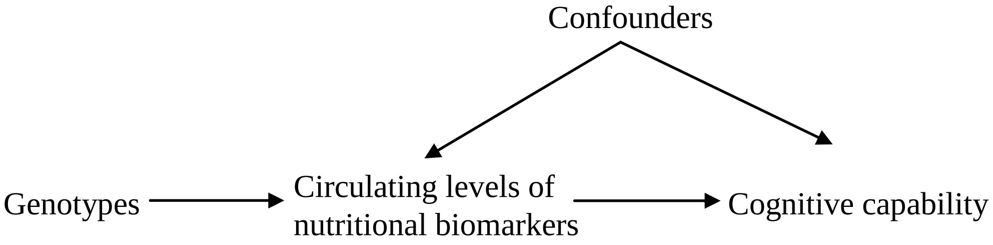

Supplemental Figure 1. Directed Acyclic Graph Representing a Genotype-Based Analysis of the Effects of Circulating Levels of Nutritional Biomarkers on Cognitive Capability

Genotypes, (e.g. SNP rs6564851) are strongly associated with a nutritional biomarker (e.g. β-carotene) but are not associated with confounders (e.g. levels of physical activity) and are not implicated in measures of cognitive capability except through their effects on the nutritional biomarker.

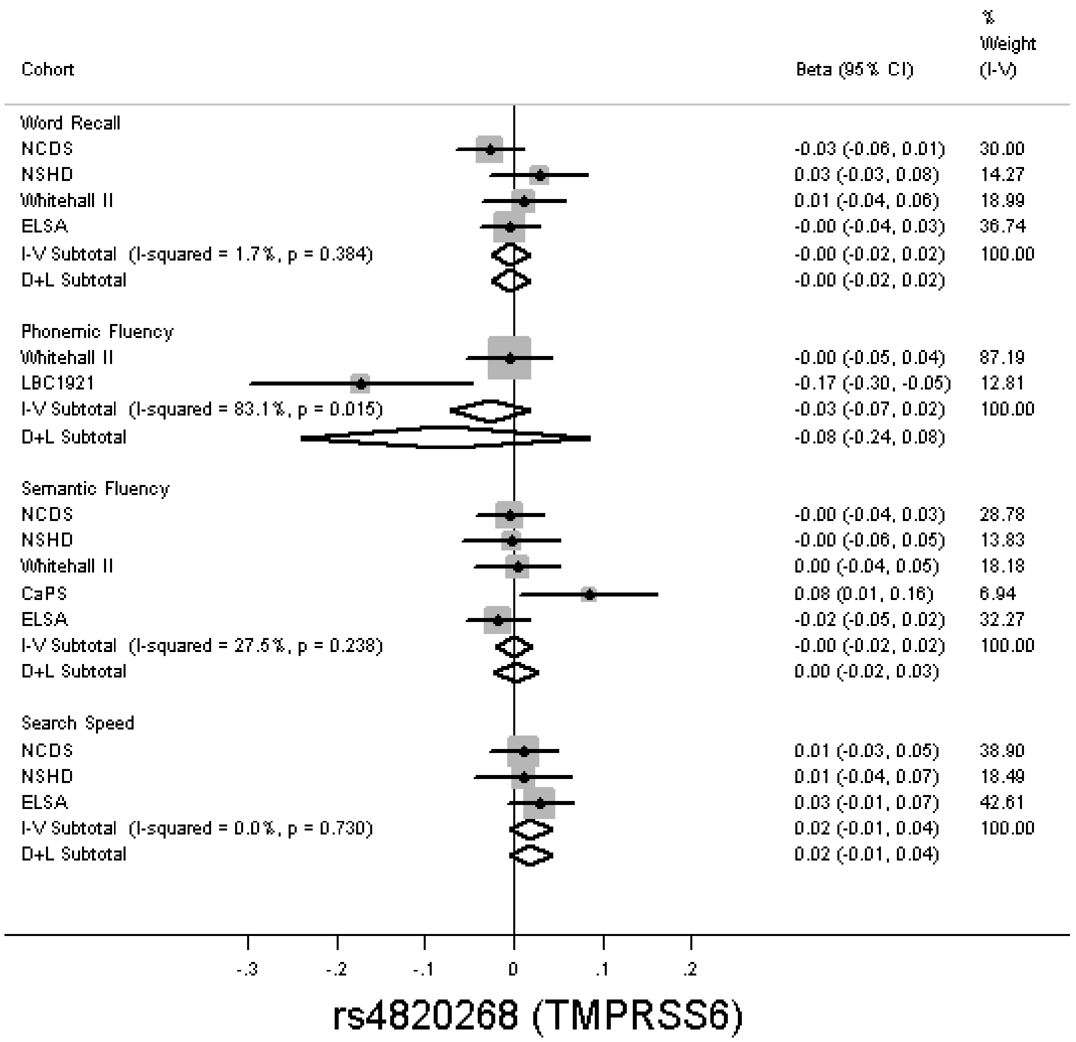

Supplemental Figure 2. Associations between Cognitive Capability Measures and rs4820268 (*TMPRSS6*) Genotype

Beta coefficients based on z-scores per A allele adjusted for age, sex, height and weight.

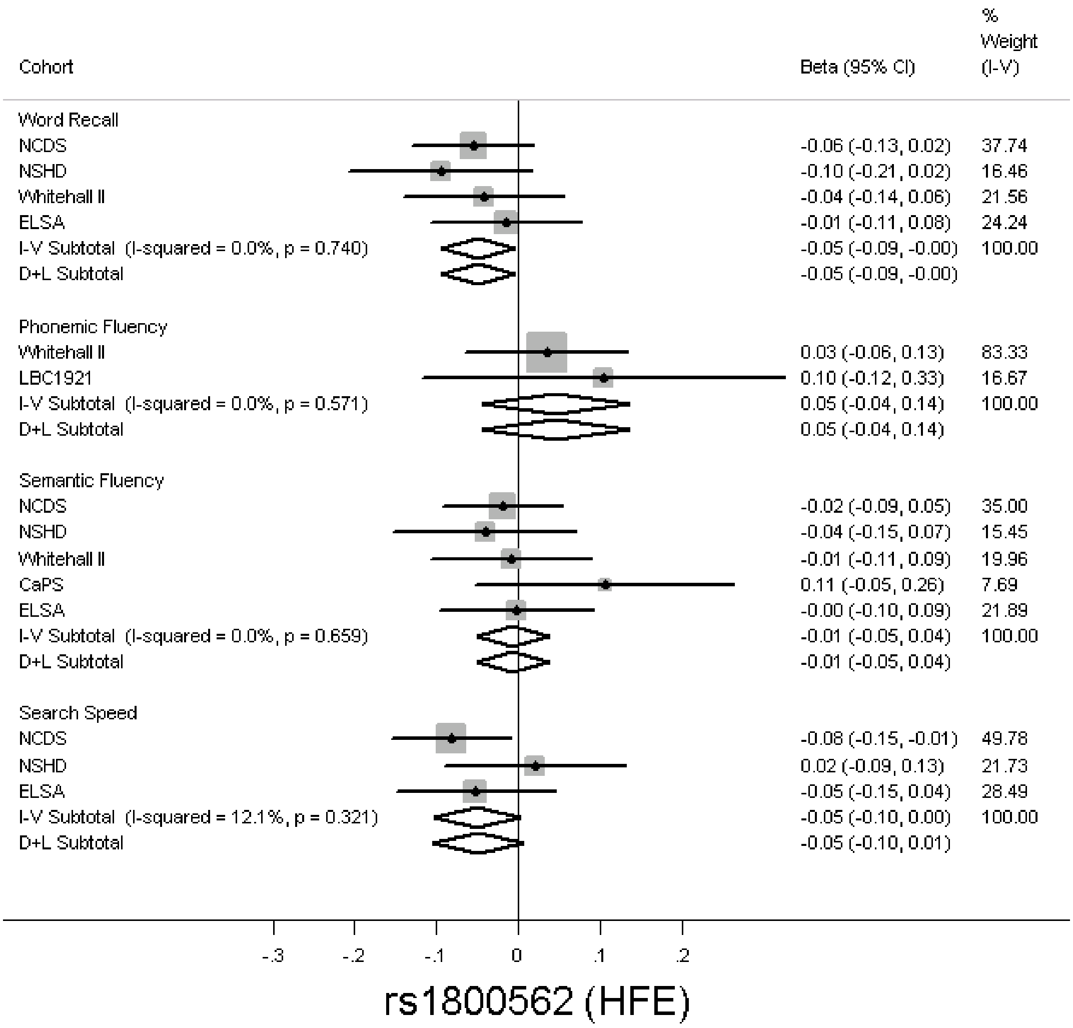

Supplemental Figure 3. Associations between Cognitive Capability Measures and rs1800562 (*HFE*) Genotype

Beta coefficients based on z-scores A/G+A/A vs G/G adjusted for age and sex.

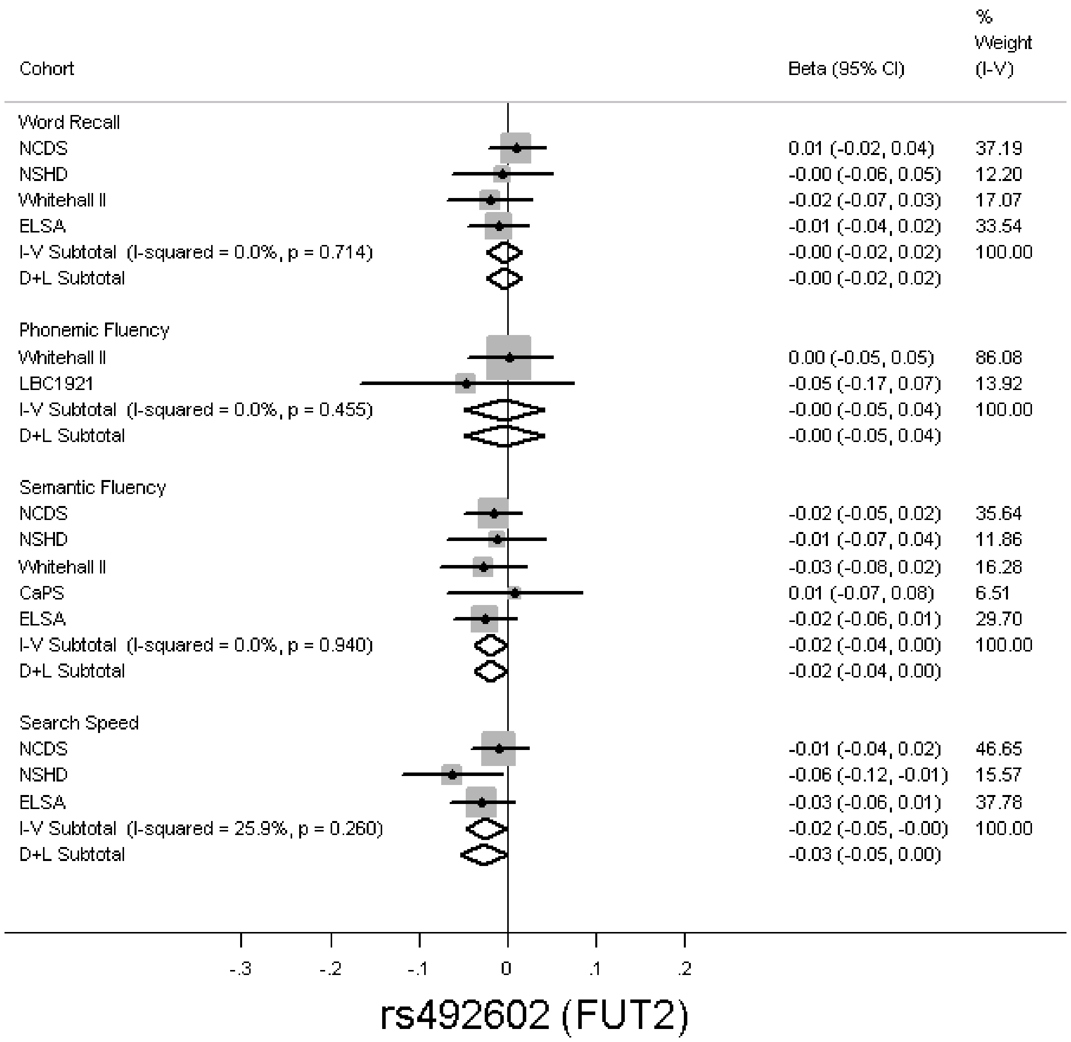

Supplemental Figure 4. Associations between Cognitive Capability Measures and rs492602 (*FUT2*) Genotype

Beta coefficients based on z-scores per G allele adjusted for age and sex.

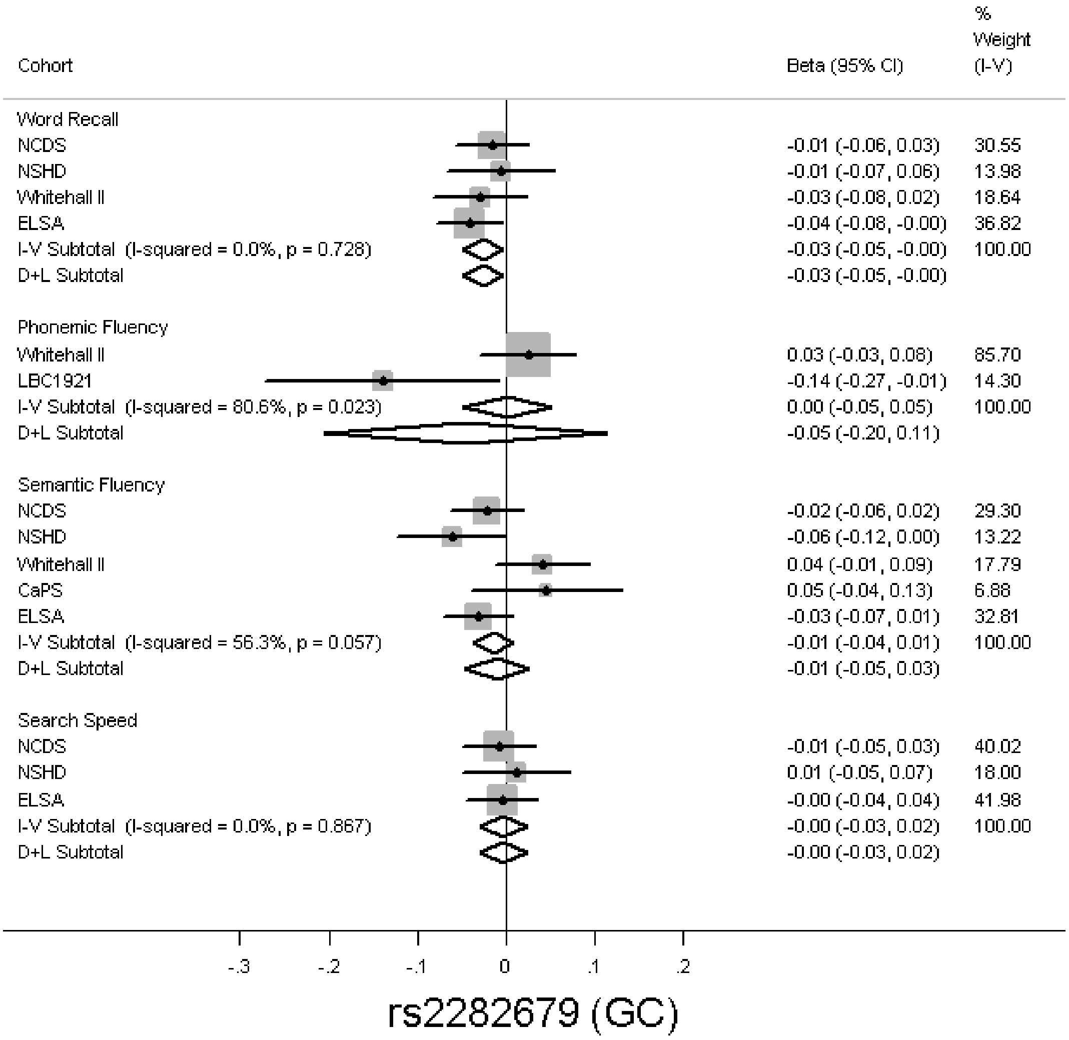

Supplemental Figure 5. Associations between Cognitive Capability Measures and rs2282679 (*GC*) Genotype

Beta coefficients based on z-scores per T allele adjusted for age and sex.

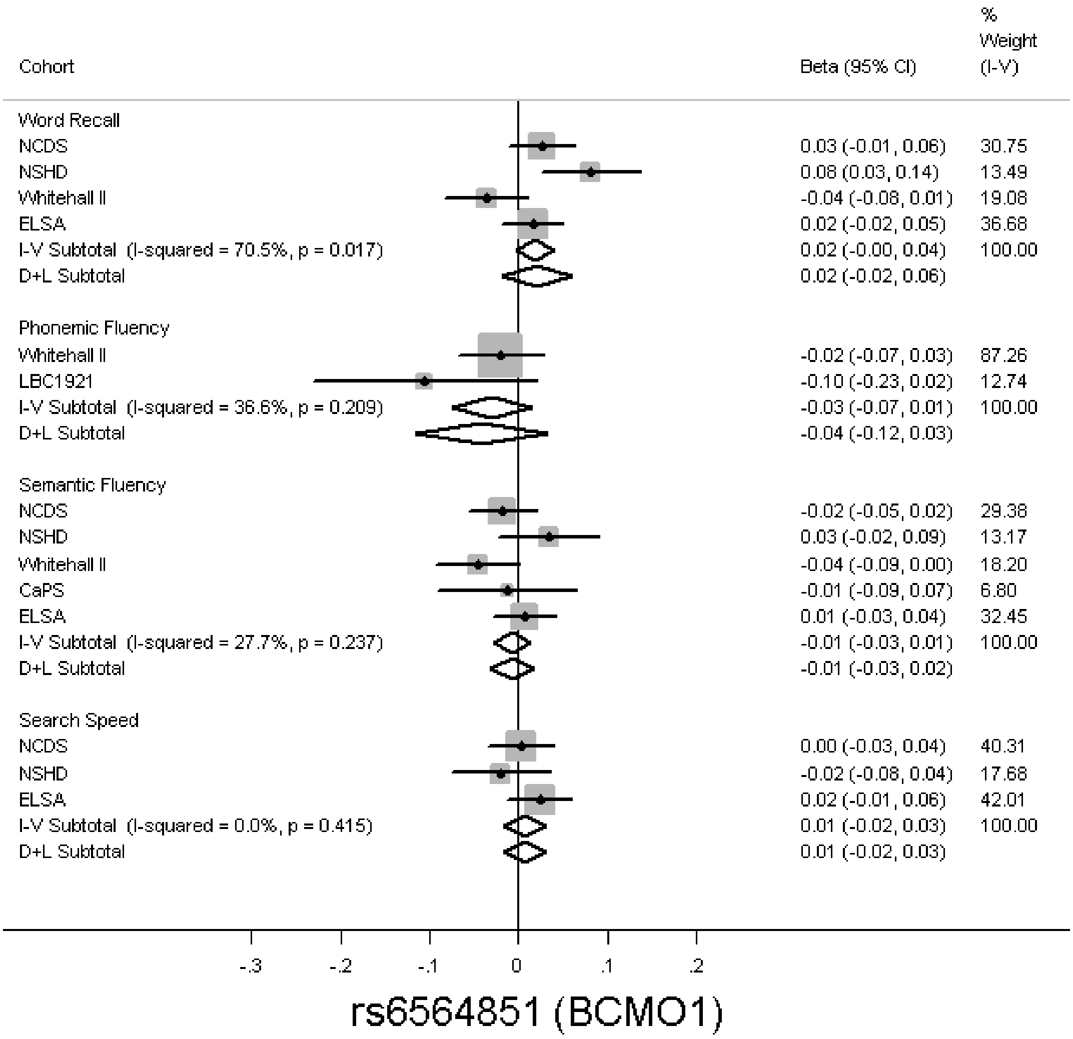

Supplemental Figure 6. Associations between Cognitive Capability Measures and rs6564851 (*BCMO1*) Genotype

Beta coefficients based on z-scores per G allele adjusted for age and sex.

Supplemental Table 1. Summary of Body Size and Demographic Characteristics by Cohort

	Cohort					
Characteristic	NCDS	NSHD	Whitehall II	CaPS	ELSA	LBC1921
Body size, mean ± sd						
Height in cm	169.3 ± 9.3	168.2 ± 8.9	172.3 ± 8.7	171.4 ± 6.4	165.7 ± 9.4	163.2 ± 9.3
Weight in kg	78.7 ± 16.3	77.5 ± 14.8	79.4 ± 13.8	78.3 ± 12.0	76.4 ± 15.3	69.9 ± 12.8
BMI in kg/m^2	27.4 ± 4.9	27.4 ± 4.7	26.7 ± 4.2	26.6 ± 3.6	27.8 ± 4.7	26.2 ± 4.1
Waist-hip ratio	0.87 ± 0.09	0.87 ± 0.09	0.91 ± 0.09	0.93 ± 0.06	0.89 ± 0.08	-
Demographics, %						
Physical activity, active	58	51	41	83	41	52
Alcohol[1], at least weekly	74	73	87	83	63	76
Smoking status, current/ex	53	71	49	82	62	56
SEP, professional/managerial	48	44	-	24	36	58

1 NSHD: more frequently than on special occasions. SEP: socio-economic position.

Supplemental Table 2. Summary of Pooled Associations between Genotypes Associated with Serum Iron and Anthropometry and Potential Confounders for Cognitive Capability

Potential Confounder	rs4820268 (*TMPRSS6*)				rs1800562 (*HFE*)			
	Beta (95% CI)	P	I^2 %; Het P	n	Beta (95% CI)	P	I^2 %; Het P	n
Height, cm	0.004 (-0.010, 0.019)	0.57	0.0; 0.9996	18093	0.014 (-0.017, 0.045)	0.38	0.0; 0.93	15790
Weight, kg	-0.022 (-0.040, -0.003)	0.0227	0.0; 0.83	18002	0.026 (-0.038, 0.089)	0.42	54.0; 0.052	15695
BMI, kg/m^2	-0.026 (-0.046, -0.005)	0.0132	0.0; 0.75	17977	0.027 (-0.049, 0.103)	0.49	61.6; 0.023	15713
WHR	-0.004 (-0.019, 0.012)	0.64	0.0; 0.96	17642	0.032 (-0.006, 0.069)	0.10	15.4; 0.32	15317
	OR (95% CI)	P	I^2 %; Het P	n/N	OR (95% CI)	P	I^2 %; Het p	n/N
Physical activity, active vs. not	0.999 (0.957, 1.043)	0.97	0.0; 0.51	8789/17711	0.927 (0.817, 1.053)	0.24	38.8; 0.15	7818/15319
Alcohol, weekly vs. less frequent	0.961 (0.915, 1.010)	0.11	0.0; 0.95	13166/17763	1.022 (0.918, 1.137)	0.67	0.0; 0.84	11759/15629
Smoking status, current/ex vs. never	1.004 (0.936, 1.076)	0.92	53.4; 0.057	10851/18205	1.063 (0.969, 1.165)	0.20	0.0; 0.74	9393/15828
SEP, professional/managerial vs. other	0.979 (0.933, 1.027)	0.39	0.0; 0.87	5901/14336	1.071 (0.947, 1.212)	0.27	21.1; 0.28	5030/12016

SEP: socio-economic position. Coefficients for rs4820268: per A allele; rs1800562: A/G+A/A vs. G/G. Models for height, weight, BMI and WHR based on z-scores. All coefficients adjusted for age and sex.

Supplemental Table 3. Summary of Pooled Associations between Genotypes Associated with Vitamin B_{12} and Vitamin D and Anthropometry and Potential Confounders for Cognitive Capability

Potential Confounder	rs492602 (*FUT2*)				rs2282679 (*GC*)			
	Beta (95% CI)	P	I^2 %; Het P	n	Beta (95% CI)	P	I^2 %; Het P	n
Height, cm	-0.007 (-0.026, 0.012)	0.45	34.3; 0.18	19908	0.015 (-0.001, 0.031)	0.07	0.0; 0.73	18150
Weight, kg	-0.011 (-0.029, 0.007)	0.24	0.0; 0.44	19782	0.004 (-0.017, 0.024)	0.72	0.0; 0.60	18063
BMI, kg/m^2	-0.007 (-0.027, 0.012)	0.45	0.0; 0.46	19780	-0.007 (-0.030, 0.015)	0.53	0.0; 0.75	18034
WHR	0.017 (-0.002, 0.035)	0.08	29.4; 0.23	19462	0.003 (-0.014, 0.020)	0.75	0.0; 0.93	17703
	OR (95% CI)	P	I^2 %; Het P	n/N	OR (95% CI)	P	I^2 %; Het P	n/N
Physical activity, active vs. not	0.972 (0.921, 1.027)	0.31	32.4; 0.19	9870/19547	1.000 (0.954, 1.049)	0.99	0.0; 0.73	8820/17776
Alcohol, weekly vs. less frequent	1.039 (0.982, 1.099)	0.19	20.6; 0.28	14449/19597	1.003 (0.950, 1.059)	0.91	0.0; 0.81	13177/17821
Smoking status, current/ex vs. never	1.030 (0.989, 1.073)	0.15	0.0; 0.91	11788/20037	1.006 (0.942, 1.075)	0.85	39.3; 0.14	10878/18265
SEP, professional/managerial vs. other	1.012 (0.967, 1.059)	0.60	0.0; 0.93	6643/15912	0.995 (0.944, 1.049)	0.87	0.0; 0.80	5927/14402

SEP: socio-economic position. Coefficients for rs492602: per G allele; rs2282679: per T allele. Models for height, weight, BMI and WHR based on z-scores. All coefficients adjusted for age and sex.

Supplemental Table 4. Summary of Pooled Associations between Genotypes Associated with β-carotene and Anthropometry and Potential Confounders for Cognitive Capability

Potential Confounder	rs6564851 (*BCMO1*)			
	Beta (95% CI)	P	I^2 %; Het P	n
Height, cm	0.007 (-0.008, 0.021)	0.36	0.0; 0.92	18069
Weight, kg	0.006 (-0.012, 0.025)	0.50	0.0; 0.76	17979
BMI, kg/m^2	0.005 (-0.015, 0.026)	0.62	0.0; 0.75	17953
WHR	0.003 (-0.012, 0.019)	0.66	0.0; 0.84	17617
	OR (95% CI)	P	I^2 %; Het P	n/N
Physical activity, active vs. not	0.976 (0.921, 1.035)	0.42	35.1; 0.17	8783/17689
Alcohol, weekly vs. less frequent	1.029 (0.980, 1.081)	0.25	0.0; 0.73	13145/17742
Smoking status, current/ex vs. never	0.987 (0.938, 1.038)	0.60	20.8; 0.28	10831/18178
SEP, professional/managerial vs. other	1.006 (0.954, 1.062)	0.82	13.0; 0.33	5888/14304

SEP: socio-economic position. Coefficients per G allele. Models for height, weight, BMI and WHR based on z-scores. All coefficients adjusted for age and sex.

Supplemental Table 5. Cognitive Capability by rs4820268 (*TMPRSS6*) Genotype and Cohort

Variable	Cohort	G/G mean ± sd [n]	A/G mean ± sd [n]	A/A mean ± sd [n]	Total mean ± sd [n]	b (95% CI) [1]	P	Heterogeneity I^2%; P
Word recall- 10 words	NCDS	6.09 ± 1.53 [1107]	6.05 ± 1.52 [2622]	6.01 ± 1.47 [1546]	6.05 ± 1.50 [5275]	-0.03 (-0.06, 0.01)	0.17	
Word recall- 45 words	NSHD	23.93 ± 6.38 [535]	23.85 ± 6.22 [1159]	24.19 ± 6.32 [689]	23.97 ± 6.28 [2383]	0.03 (-0.03, 0.08)	0.32	
Word recall- 20 words	Whitehall II	7.02 ± 2.40 [678]	6.95 ± 2.36 [1536]	7.13 ± 2.47 [909]	7.02 ± 2.40 [3123]	0.01 (-0.04, 0.06)	0.66	
Word recall- 10 words	ELSA	5.00 ± 1.66 [1136]	5.00 ± 1.68 [2542]	4.99 ± 1.69 [1575]	5.00 ± 1.68 [5253]	-0.00 (-0.04, 0.03)	0.81	
	**Pooled**	**[3456]**	**[7859]**	**[4719]**	**[16034]**	**-0.003 (-0.024, 0.018)**	**0.75**	**1.7; 0.38**
Phonemic fluency- 1 letter	Whitehall II	16.09 ± 3.97 [676]	15.95 ± 4.06 [1536]	16.14 ± 4.24 [901]	16.03 ± 4.10 [3113]	-0.00 (-0.05, 0.04)	0.84	
Phonemic fluency- 3 letters	LBC1921	42.98 ± 11.25 [84]	40.40 ± 12.39 [262]	38.60 ± 12.20 [163]	40.25 ± 12.21 [509]	-0.17 (-0.30, -0.05)	0.0076	
	**Pooled**	**[760]**	**[1798]**	**[1064]**	**[3622]**	**-0.078 (-0.240, 0.085)**	**0.35**	**83.1; 0.015**
Semantic fluency	NCDS	22.5 ± 6.4 [1113]	22.5 ± 6.4 [2640]	22.5 ± 6.1 [1553]	22.5 ± 6.3 [5306]	-0.00 (-0.04, 0.03)	0.80	
	NSHD	23.7 ± 7.0 [549]	23.7 ± 6.7 [1174]	23.7 ± 6.8 [702]	23.7 ± 6.8 [2425]	-0.00 (-0.06, 0.05)	0.92	
	Whitehall II	16.0 ± 3.7 [678]	16.0 ± 3.6 [1541]	16.1 ± 4.0 [904]	16.0 ± 3.7 [3123]	0.00 (-0.04, 0.05)	0.89	
	CaPS	16.2 ± 4.7 [278]	16.5 ± 4.9 [593]	17.0 ± 4.6 [329]	16.6 ± 4.8 [1200]	0.08 (0.01, 0.16)	0.0331	
	ELSA	20.4 ± 6.1 [1137]	20.3 ± 6.2 [2545]	20.2 ± 6.1 [1575]	20.3 ± 6.1 [5257]	-0.02 (-0.05, 0.02)	0.35	
	**Pooled**	**[3755]**	**[8493]**	**[5063]**	**[17311]**	**0.002 (-0.023, 0.026)**	**0.90**	**27.5; 0.24**
Search speed-780 letters	NCDS	332 ± 83 [1088]	334 ± 86 [2594]	334 ± 89 [1522]	333 ± 86 [5204]	0.01 (-0.03, 0.05)	0.60	

Search speed-600 letters	NSHD	283 ± 76 [543]	281 ± 76 [1171]	283 ± 77 [699]	282 ± 76 [2413]	0.01 (-0.04, 0.07)	0.70	
Search speed-780 letters	ELSA	298 ± 89 [1126]	299 ± 89 [2520]	304 ± 91 [1558]	300 ± 89 [5204]	0.03 (-0.01, 0.07)	0.11	
	**Pooled**	**[2757]**	**[6285]**	**[3779]**	**[12821]**	**0.019 (-0.005, 0.042)**	**0.12**	**0.0; 0.73**

1: Beta coefficients based on z-scores per A allele adjusted for age, sex, height and weight.

Supplemental Table 6. Cognitive Capability by rs1800562 (*HFE*) Genotype and Cohort

Variable	Cohort	G/G mean ± sd [n]	A/G+A/A mean ± sd [n]	Total mean ± sd [n]	b (95% CI) [1]	P	Heterogeneity I^2%; P
Word recall- 10 words	NCDS	6.06 ± 1.51 [4617]	5.96 ± 1.48 [822]	6.04 ± 1.50 [5439]	-0.06 (-0.13, 0.02)	0.14	
Word recall- 45 words	NSHD	24.04 ± 6.28 [2050]	23.46 ± 6.49 [354]	23.95 ± 6.31 [2404]	-0.10 (-0.21, 0.02)	0.095	
Word recall- 20 words	Whitehall II	7.03 ± 2.41 [2699]	6.89 ± 2.34 [436]	7.01 ± 2.40 [3135]	-0.04 (-0.14, 0.06)	0.40	
Word recall- 10 words	ELSA	4.87 ± 1.62 [2690]	4.87 ± 1.51 [437]	4.87 ± 1.60 [3127]	-0.01 (-0.11, 0.08)	0.76	
	**Pooled**	**[12056]**	**[2049]**	**[14105]**	**-0.049 (-0.095, -0.004)**	**0.033**	**0.0; 0.74**
Phonemic fluency- 1 letter	Whitehall II	16.01 ± 4.14 [2690]	16.09 ± 3.88 [435]	16.02 ± 4.10 [3125]	0.03 (-0.06, 0.13)	0.50	
Phonemic fluency- 3 letters	LBC1921	39.88 ± 12.25 [416]	41.15 ± 12.52 [97]	40.12 ± 12.30 [513]	0.10 (-0.12, 0.33)	0.36	
	**Pooled**	**[3106]**	**[532]**	**[3638]**	**0.046 (-0.044, 0.136)**	**0.32**	**0.0; 0.57**
	NCDS	22.5 ± 6.3 [4645]	22.4 ± 6.3 [827]	22.5 ± 6.3 [5472]	-0.02 (-0.09, 0.05)	0.61	
	NSHD	23.7 ± 6.9 [2088]	23.4 ± 6.1 [358]	23.7 ± 6.8 [2446]	-0.04 (-0.15, 0.07)	0.47	
Semantic fluency	Whitehall II	16.0 ± 3.8 [2699]	15.9 ± 3.6 [436]	16.0 ± 3.7 [3135]	-0.01 (-0.11, 0.09)	0.86	
	CaPS	16.4 ± 4.7 [1037]	17.0 ± 4.9 [173]	16.5 ± 4.8 [1210]	0.11 (-0.05, 0.26)	0.19	
	ELSA	19.9 ± 5.8 [2691]	19.9 ± 5.5 [438]	19.9 ± 5.8 [3129]	-0.00 (-0.10, 0.09)	0.95	
	**Pooled**	**[13160]**	**[2232]**	**[15392]**	**-0.007 (-0.051, 0.036)**	**0.74**	**0.0; 0.66**
Search speed-780 letters	NCDS	334 ± 87 [4556]	327 ± 82 [809]	333 ± 86 [5365]	-0.08 (-0.15, -0.01)	0.029	

Search speed-600 letters	NSHD	282 ± 76 [2075]	284 ± 79 [358]	282 ± 76 [2433]	0.02 (-0.09, 0.13)	0.72	
Search speed-780 letters	ELSA	298 ± 90 [2672]	295 ± 83 [440]	298 ± 89 [3112]	-0.05 (-0.15, 0.04)	0.29	
	**Pooled**	**[9303]**	**[1607]**	**[10910]**	**-0.049 (-0.105, 0.006)**	**0.08**	**12.1; 0.32**

1: Beta coefficients based on z-scores A/G+A/A vs G/G adjusted for age and sex.

Supplemental Table 7. Cognitive Capability by rs492602 (*FUT2*) Genotype and Cohort

Variable	Cohort	A/A mean ± sd [n]	A/G mean ± sd [n]	G/G mean ± sd [n]	Total mean ± sd [n]	b (95% CI) [1]	P	Heterogeneity I^2%; P
Word recall- 10 words	NCDS	6.02 ± 1.53 [1761]	6.01 ± 1.50 [3644]	6.05 ± 1.49 [1864]	6.02 ± 1.50 [7269]	0.01 (-0.02, 0.04)	0.50	
Word recall- 45 words	NSHD	23.65 ± 6.42 [573]	24.26 ± 6.23 [1220]	23.61 ± 6.36 [608]	23.95 ± 6.31 [2401]	-0.00 (-0.06, 0.05)†	0.87	
Word recall- 20 words	Whitehall II	7.00 ± 2.35 [758]	7.03 ± 2.37 [1566]	7.00 ± 2.51 [812]	7.01 ± 2.40 [3136]	-0.02 (-0.07, 0.03)	0.42	
Word recall- 10 words	ELSA	5.00 ± 1.68 [1391]	4.92 ± 1.71 [2730]	4.99 ± 1.66 [1368]	4.96 ± 1.69 [5489]	-0.01 (-0.04, 0.02)	0.56	
	**Pooled**	**[4483]**	**[9160]**	**[4652]**	**[18295]**	**-0.003 (-0.023, 0.017)**	**0.75**	**0.0; 0.71**
Phonemic fluency- 1 letter	Whitehall II	15.90 ± 4.11 [758]	16.06 ± 4.11 [1559]	16.07 ± 4.07 [809]	16.02 ± 4.10 [3126]	0.00 (-0.05, 0.05)	0.89	
Phonemic fluency- 3 letters	LBC1921	41.10 ± 12.33 [105]	39.92 ± 12.49 [239]	39.80 ± 12.04 [169]	40.12 ± 12.30 [513]	-0.05 (-0.17, 0.07)	0.45	
	**Pooled**	**[863]**	**[1798]**	**[978]**	**[3639]**	**-0.004 (-0.048, 0.041)**	**0.88**	**0.0; 0.45**
	NCDS	22.6 ± 6.4 [1778]	22.4 ± 6.3 [3662]	22.4 ± 6.3 [1877]	22.5 ± 6.3 [7317]	-0.02 (-0.05, 0.02)	0.34	
	NSHD	23.7 ± 6.6 [580]	23.7 ± 6.9 [1244]	23.5 ± 6.9 [619]	23.7 ± 6.8 [2443]	-0.01 (-0.07, 0.04)	0.67	

Semantic fluency	Whitehall II	15.9 ± 3.7 [759]	16.2 ± 3.7 [1566]	15.8 ± 3.8 [811]	16.0 ± 3.7 [3136]	-0.03 (-0.08, 0.02)†	0.27	
	CaPS	16.4 ± 4.8 [310]	16.6 ± 4.7 [572]	16.4 ± 4.8 [328]	16.5 ± 4.7 [1210]	0.01 (-0.07, 0.08)	0.84	
	ELSA	20.4 ± 6.2 [1393]	20.1 ± 6.3 [2732]	20.2 ± 5.9 [1368]	20.2 ± 6.1 [5493]	-0.02 (-0.06, 0.01)	0.17	
	**Pooled**	**[4820]**	**[9776]**	**[5003]**	**[19599]**	**-0.018 (-0.038, 0.001)**	**0.064**	**0.0; 0.94**
Search speed-780 letters	NCDS	333 ± 85 [1753]	335 ± 89 [3586]	331 ± 84 [1844]	333 ± 87 [7183]	-0.01 (-0.04, 0.02)	0.60	
Search speed-600 letters	NSHD	285 ± 81 [576]	284 ± 76 [1239]	275 ± 70 [615]	282 ± 76 [2430]	-0.06 (-0.12, -0.01)	0.030	
Search speed-780 letters	ELSA	304 ± 93 [1376]	296 ± 88 [2686]	299 ± 90 [1364]	299 ± 90 [5426]	-0.03 (-0.06, 0.01)†	0.13	
	**Pooled**	**[3705]**	**[7511]**	**[3823]**	**[15039]**	**-0.026 (-0.052, 0.000)**	**0.053**	**25.9; 0.26**

†Full genotype model representing a significantly better fit than the given per allele model.
1: Beta coefficients based on z-scores per G allele adjusted for age and sex.

Supplemental Table 8. Cognitive Capability by rs2282679 (*GC*) Genotype and Cohort

Variable	Cohort	G/G mean ± sd [n]	G/T mean ± sd [n]	T/T mean ± sd [n]	Total mean ± sd [n]	b (95% CI) [1]	P	Heterogeneity I^2%; P
Word recall- 10 words	NCDS	6.14 ± 1.50 [457]	6.02 ± 1.45 [2265]	6.04 ± 1.54 [2702]	6.04 ± 1.50 [5424]	-0.01 (-0.06, 0.03)	0.49	
Word recall- 45 words	NSHD	23.57 ± 6.53 [194]	24.21 ± 6.19 [991]	23.83 ± 6.27 [1290]	23.96 ± 6.26 [2475]	-0.01 (-0.07, 0.06)	0.85	
Word recall- 20 words	Whitehall II	7.11 ± 2.27 [259]	7.04 ± 2.38 [1313]	6.97 ± 2.43 [1551]	7.01 ± 2.39 [3123]	-0.03 (-0.08, 0.02)	0.29	
Word recall- 10 words	ELSA	4.99 ± 1.63 [463]	5.01 ± 1.70 [2249]	4.92 ± 1.68 [2793]	4.96 ± 1.69 [5505]	-0.04 (-0.08, -0.00)	0.034	
	**Pooled**	**[1373]**	**[6818]**	**[8336]**	**[16527]**	**-0.026 (-0.048, -0.003)**	**0.028**	**0.0; 0.73**

Phonemic fluency- 1 letter	Whitehall II	16.02 ± 3.98 [257]	15.93 ± 4.18 [1311]	16.11 ± 4.07 [1546]	16.03 ± 4.11 [3114]	0.03 (-0.03, 0.08)	0.35	
Phonemic fluency- 3 letters	LBC1921	43.87 ± 13.69 [47]	40.33 ± 11.53 [203]	39.44 ± 12.41 [261]	40.20 ± 12.23 [511]	-0.14 (-0.27, -0.01)	0.039	
	**Pooled**	**[304]**	**[1514]**	**[1807]**	**[3625]**	**-0.045 (-0.205, 0.114)**	**0.58**	**80.6; 0.023**
	NCDS	22.8 ± 6.0 [459]	22.5 ± 6.3 [2281]	22.4 ± 6.3 [2717]	22.5 ± 6.3 [5457]	-0.02 (-0.06, 0.02)	0.32	
	NSHD	24.5 ± 7.6 [197]	23.7 ± 6.7 [1012]	23.4 ± 6.9 [1314]	23.6 ± 6.9 [2523]	-0.06 (-0.12, 0.00)	0.052	
Semantic fluency	Whitehall II	15.8 ± 3.8 [259]	15.9 ± 3.8 [1313]	16.1 ± 3.7 [1551]	16.0 ± 3.7 [3123]	0.04 (-0.01, 0.09)	0.13	
	CaPS	16.0 ± 4.8 [105]	16.6 ± 4.8 [480]	16.6 ± 4.7 [630]	16.6 ± 4.8 [1215]	0.05 (-0.04, 0.13)	0.29	
	ELSA	20.3 ± 6.3 [463]	20.3 ± 6.1 [2249]	20.1 ± 6.2 [2797]	20.2 ± 6.1 [5509]	-0.03 (-0.07, 0.01)	0.12	
	**Pooled**	**[1483]**	**[7335]**	**[9009]**	**[17 827]**	**-0.010 (-0.046, 0.026)**	**0.59**	**56.3; 0.057**
Search speed-780 letters	NCDS	327 ± 77 [450]	336 ± 88 [2237]	332 ± 86 [2666]	333 ± 86 [5353]	-0.01 (-0.05, 0.03)†	0.72	
Search speed-600 letters	NSHD	281 ± 71 [196]	281 ± 75 [1008]	282 ± 78 [1307]	282 ± 76 [2511]	0.01 (-0.05, 0.07)	0.70	
Search speed-780 letters	ELSA	300 ± 91 [454]	299 ± 90 [2228]	299 ± 91 [2759]	299 ± 90 [5441]	-0.00 (-0.04, 0.04)	0.83	
	**Pooled**	**[1100]**	**[5473]**	**[6732]**	**[13 305]**	**-0.003 (-0.029, 0.023)**	**0.84**	**0.0; 0.87**

†Full genotype model representing a significantly better fit than the given per allele model.
1: Beta coefficients based on z-scores per T allele adjusted for age and sex.

Supplemental Table 9. Cognitive Capability by rs6564851 (*BCMO1*) Genotype and Cohort

Variable	Cohort	T/T mean ± sd [n]	G/T mean ± sd [n]	G/G mean ± sd [n]	Total mean ± sd [n]	b (95% CI) [1]	P	Heterogeneity I^2%; P
Word recall- 10 words	NCDS	5.97 ± 1.53 [1208]	6.06 ± 1.50 [2675]	6.06 ± 1.48 [1552]	6.04 ± 1.50 [5435]	0.03 (-0.01, 0.06)	0.15	
Word recall- 45 words	NSHD	23.43 ± 6.17 [482]	23.86 ± 6.35 [1183]	24.46 ± 6.32 [735]	23.96 ± 6.31 [2400]	0.08 (0.03, 0.14)	0.0042	
Word recall- 20 words	Whitehall II	7.17 ± 2.40 [691]	6.96 ± 2.44 [1519]	6.98 ± 2.33 [925]	7.01 ± 2.40 [3135]	-0.04 (-0.08, 0.01)	0.14	
Word recall- 10 words	ELSA	4.95 ± 1.69 [1213]	4.96 ± 1.69 [2692]	4.96 ± 1.69 [1583]	4.96 ± 1.69 [5488]	0.02 (-0.02, 0.05)	0.33	
	**Pooled**	**[3594]**	**[8069]**	**[4795]**	**[16458]**	**0.021 (-0.018, 0.060)**	**0.29**	**70.5; 0.017**
Phonemic fluency- 1 letter	Whitehall II	16.10 ± 3.88 [690]	16.04 ± 4.25 [1513]	15.95 ± 4.02 [922]	16.03 ± 4.10 [3125]	-0.02 (-0.07, 0.03)	0.44	
Phonemic fluency- 3 letters	LBC1921	42.10 ± 13.52 [91]	39.95 ± 11.95 [259]	39.29 ± 12.09 [163]	40.12 ± 12.30 [513]	-0.10 (-0.23, 0.02)	0.10	
	**Pooled**	**[781]**	**[1772]**	**[1085]**	**[3638]**	**-0.042 (-0.116, 0.033)**	**0.27**	**36.6; 0.21**
Semantic fluency	NCDS	22.7 ± 6.4 [1216]	22.4 ± 6.3 [2691]	22.5 ± 6.2 [1561]	22.5 ± 6.3 [5468]	-0.02 (-0.05, 0.02)	0.37	
	NSHD	23.7 ± 6.6 [495]	23.4 ± 6.8 [1201]	24.1 ± 6.9 [746]	23.7 ± 6.8 [2442]	0.03 (-0.02, 0.09)	0.22	
	Whitehall II	16.3 ± 3.7 [693]	15.9 ± 3.8 [1517]	16.0 ± 3.7 [925]	16.0 ± 3.7 [3135]	-0.04 (-0.09, 0.00)	0.064	
	CaPS	16.4 ± 5.1 [264]	16.7 ± 4.8 [578]	16.3 ± 4.5 [362]	16.5 ± 4.8 [1204]	-0.01 (-0.09, 0.07)	0.76	
	ELSA	20.3 ± 6.2 [1214]	20.0 ± 6.1 [2695]	20.3 ± 6.1 [1583]	20.2 ± 6.1 [5492]	0.01 (-0.03, 0.04)	0.68	
	**Pooled**	**[3882]**	**[8682]**	**[5177]**	**[17741]**	**-0.007 (-0.032, 0.017)**	**0.57**	**27.7; 0.24**
Search speed-780 letters	NCDS	332 ± 88 [1194]	334 ± 88 [2633]	333 ± 82 [1534]	333 ± 86 [5361]	0.00 (-0.03, 0.04)	0.85	

Search speed-600 letters	NSHD	282 ± 77 [492]	283 ± 75 [1195]	280 ± 78 [742]	282 ± 76 [2429]	-0.02 (-0.08, 0.04)	0.49	
Search speed-780 letters	ELSA	298 ± 88 [1198]	298 ± 91 [2659]	300 ± 92 [1566]	299 ± 91 [5423]	0.02 (-0.01, 0.06)	0.19	
	**Pooled**	**[2884]**	**[6487]**	**[3842]**	**[13213]**	**0.008 (-0.015, 0.031)**	**0.50**	**0.0; 0.42**

1: Beta coefficients based on z-scores per G allele adjusted for age and sex.
